# A Machine-Learning-Based Analysis of Resting State Electroencephalogram Signals to Identify Latent Schizotypal and Bipolar Development in Healthy University Students

**DOI:** 10.3390/diagnostics15040454

**Published:** 2025-02-13

**Authors:** Flórián Gubics, Ádám Nagy, József Dombi, Antónia Pálfi, Zoltán Szabó, Zsolt János Viharos, Anh Tuan Hoang, Vilmos Bilicki, István Szendi

**Affiliations:** 1 Department of Medical Genetics, Doctoral School of Interdisciplinary Medicine, University of Szeged, 6720 Szeged, Hungary; gubics.florian@gmail.com; 2 Department of Software Engineering, University of Szeged, 6720 Szeged, Hungary; 3 Department of Computer Algorithms and Artificial Intelligence, University of Szeged, Árpád Square 2, 6720 Szeged, Hungary; 4 HUN-REN-SZTE Research Group on Artificial Intelligence, Institute of Informatics, University of Szeged, Tisza Lajos Boulevard 103, 6725 Szeged, Hungary; 5 HUN-REN Institute for Computer Science and Control (SZTAKI), Center of Excellence in Production Informatics and Control, Centre of Excellence of the Hungarian Academy of Sciences (MTA), Kende Street 13-17, H-1111 Budapest, Hungary; 6 Faculty of Economics and Business, John von Neumann University, Izsák Street 10, 6400 Kecskemét, Hungary; 7 Department of Psychiatry, Kiskunhalas Semmelweis Hospital, Dr. Monszpart László Street 1, 6400 Kiskunhalas, Hungary; 8 Department of Clinical- and Health Psychology, Institute of Psychology, University of Szeged, Egyetem Street 2, 6720 Szeged, Hungary; 9 Centre of Excellence for Interdisciplinary Research, Development and Innovation, University of Szeged, Dugonics Square 13, 6720 Szeged, Hungary

**Keywords:** psychology, signal processing, analysis, bipolar depression, schizophrenia, early detection

## Abstract

**Background**: Early and accurate diagnosis is crucial for effective prevention and treatment of severe mental illnesses, such as schizophrenia and bipolar disorder. However, identifying these conditions in their early stages remains a significant challenge. Our goal was to develop a method capable of detecting latent disease liability in healthy volunteers. **Methods**: Using questionnaires examining affective temperament and schizotypal traits among voluntary, healthy university students (N = 710), we created three groups. These were a group characterized by an emphasis on positive schizotypal traits (N = 20), a group showing cyclothymic temperament traits (N = 17), and a control group showing no susceptibility in either direction (N = 21). We performed a resting-state EEG examination as part of a complex psychological, electrophysiological, psychophysiological, and laboratory battery, and we developed feature-selection machine-learning methods to differentiate the low-risk groups. **Results**: Both low-risk groups could be reliably (with 90% accuracy) separated from the control group. **Conclusions**: Models applied to the data allowed us to differentiate between healthy university students with latent schizotypal or bipolar tendencies. Our research may improve the sensitivity and specificity of risk-state identification, leading to more effective and safer secondary prevention strategies for individuals in the prodromal phases of these disorders.

## 1. Introduction

With sophisticated and consistent prevention methods applied before the manifestation of schizophrenia–bipolar spectrum diseases of neurodevelopmental origin, typically in young adulthood, the development can sometimes be directed in a neurotypical direction to prevent the diseases. For this, we would need to reliably identify persons at risk. Resting-state electroencephalogram (rsEEG) is a simple non-invasive diagnostic procedure widely used in healthcare, but its traditional evaluation method does not help us to diagnose advanced disease states. Could the additional information obtained with machine-learning methods be enough to be widely used in the early detection and prevention of mental disorders? Schizophrenia (SZ) and bipolar disorder (BD) are serious mental illnesses; their combined lifetime prevalence exceeds 5% [1,2]. Both diseases severely affect quality of life, ability to work, and participation in society and are associated with a very high risk of suicide [3,4].

The diagnosis of psychiatric disorders typically relies on unstructured or semi-structured interviews in both clinical practice and research settings since prodromal self-experience disorders can be approached with a phenomenological interview that is different from traditional psychiatric interviews and requires special training. However, this approach lacks precision due to the heterogeneous nature of symptoms and the overlap between related neurodevelopmental disorders, while its scalability remains insufficient. As a result, late or incorrect diagnoses are common, highlighting the need to enhance traditional diagnostic procedures with objective, instrumental data. Such data can refine the characterization of examined phenotypes and have the potential to help detect subtle, early-stage changes that may precede the manifestation of overt clinical symptoms. This early detection capability could improve the accuracy of current diagnostic practices, enable timely interventions, and support personalized treatment strategies, ultimately enhancing patient outcomes and disease management. Furthermore, these advancements can contribute to a deeper understanding of certain mental illnesses [5,6,7].

Recognizing pre-disease conditions to effectively indicated preventive interventions presents a significant challenge. Psychosis spectrum disorders (which encompass conditions such as schizophrenia and bipolar disorder), which usually manifest during adolescence or young adulthood as psychosis or major affective episodes, are generally lifelong, chronic, and irreversible. However, these disorders are often preceded by prodromal states that can last several years, during which targeted interventions may redirect neurodevelopment towards typical pathways, thus preventing the full manifestation and progression of the disease. Enhancing the detection of subtle and elusive changes in experience and behavior during the prodrome is crucial. This enhancement can be achieved by incorporating instrumental diagnostic procedures that help identify these early changes. Therefore, defining biomarkers that facilitate the prompt and accurate identification of at-risk individuals is essential [8,9,10].

Advances in neuroimaging and artificial intelligence (AI) are increasingly aiding our understanding and diagnosis of these conditions. Among brain imaging procedures, EEG is gaining wider acceptance in hospitals and outpatient clinics, as it is more cost-effective and simpler to implement, which justifies its application and preference in research. Although traditional EEG analysis has not been part of psychiatric diagnostics for decades, AI-driven models, including machine-learning algorithms from data extracted via EEG, are now providing essential support in detecting psychiatric diseases and differentiating them from each other and healthy individuals [6,7,11,12].

EEG is useful for obtaining a more nuanced understanding of brain function by recording brain surface electrical signals [13,14]. In clinical trials, EEG is typically applied in three ways: frequency analysis, microstate (MS) analysis, and event-related potential (ERP) analysis. Brain activity can be categorized into frequency bands such as delta (below 4 Hz), theta (4–8 Hz), alpha (8–13 Hz), beta (14–30 Hz), and gamma (above 30 Hz), which are analyzed through frequency analysis [15,16]. Frequency analysis dominates the study of psychiatric disorders, particularly schizophrenia; however, the research literature on bipolar disorder within the psychosis spectrum is notably less extensive. Addressing the inconsistencies among study results remains a challenge, with more effective standardization of methodologies and multi-channel analyses across several brain regions providing potential solutions [11]. MS involves short-term electrical state changes that span multiple brain areas. This method offers a comprehensive view of the brain’s network system, and it has significant potential in investigating both schizophrenia and bipolar disorder [14,17,18,19,20,21]. Both frequency and microstate analyses can be conducted using an rsEEG setup, in which participants remain in a relaxed state with their eyes either open or closed, allowing the device to measure spontaneous electrical activity. This setup is commonly used in the investigation of psychiatric disorders, as it is easier to implement compared to task-based paradigms typically required for ERP analysis [16]. Within rsEEG protocols, the eyes-closed condition is the most frequently employed in psychiatric research. Additionally, the eyes-closed condition results in less noisy data by minimizing external stimuli, which facilitates the analysis of inherently complex rsEEG data. Furthermore, it provides an optimal framework for enhanced alpha frequency activity, which is an important indicator of changes in cognitive functioning, a key symptom in the early stages of both disorders [9,11,15,21,22]. Considering these factors, the present study utilizes the rsEEG eyes-closed condition and works with data obtained through frequency and microstate analysis.

The analysis employs innovative machine-learning frameworks for feature selection and model explainability, specifically Adaptive Hybrid Feature Selection (AHFS) [23] and Clique Forming Feature Selection (CFFS) [24]. AHFS is a novel feature selection algorithm that adaptively combines correlation-based and information-theoretic methods in a single framework, outperforming traditional fixed-criterion approaches. By dynamically exploring both feature and evaluation spaces, it delivers higher accuracy and robustness, especially in high-dimensional, noisy datasets. Meanwhile, CFFS uniquely employs a model-agnostic feature-importance calculation (Shapley values) aggregated across multiple ML models. Those models’ feature sets were formed in a way to reduce inter-correlation among features, countering a key weakness of Shapley values. Leveraging standard cross-validation for noise minimization, CFFS identifies a broader set of globally important features, complementing the more compact subsets found by AHFS for robust, efficient feature selection.

In this study, we focus on the premorbid period that precedes the prodrome, where only latent susceptibility characteristics are observable among healthy, symptom-free young individuals. This cohort is very important as, in the case of psychiatric disorders, there is a research gap in the development of identification methods for the premorbid state [25]. Our objective was to distinguish between healthy university students who exhibit a potential latent predisposition toward schizotypal or bipolar disorders and those without such susceptibilities, utilizing rsEEG data for this analysis. In addition, it aims to differentiate between the two groups of susceptibilities. The challenge of identifying risk conditions of varying severity is significant. This challenge is further compounded by the limited number of subjects typically available at general clinical and hospital research sites. Consequently, our methodological development needed to ensure that the robust machine-learning methods used for model explanation were also suitable for analyzing small datasets, while being sensitive enough to accurately detect the target population.

## 2. State of the Art

The application of AI in the research field provides a significant opportunity to aid the analysis of EEG data. Typically, this involves transforming EEG frequency data into unique characteristics through mathematical processes, which are then used to create models. These models have the potential to assist in distinguishing different groups with greater accuracy and help in diagnostic and differential diagnostic tasks [26,27,28,29]. The models and features developed through AI methodologies provide a deeper and more complex analysis of the electrophysiological markers of schizophrenia and bipolar disorder. However, challenges remain due to the lack of transparency [30], and also standardization in methodologies and analyses [6,7,26].

Given that the literature offers a wide range of methods and approaches for investigating psychiatric disorders using AI and EEG, including the study of schizophrenia and bipolar disorder, this introduction highlights a selection of examples that illustrate commonly used tools and the accuracy achievable with these procedures, as well as the number of participants in relevant studies (which is not uncommonly low). However, more comprehensive information on numerous other examples can be found in review articles, which provide detailed insights into current approaches and potential future directions [6,7,27,28]. Table 1 presents relevant studies along with the methods applied.

Machine-learning (ML) and deep-learning (DL) techniques play a crucial role in classification and prediction. Support Vector Machines (SVMs) are widely used for their effectiveness in distinguishing psychiatric conditions, while Decision Trees and Random Forests offer interpretability for clinical applications. Deep-learning approaches, such as Convolutional Neural Networks (CNNs), excel in extracting complex EEG features but require extensive data and computational resources [7,27]. The selection and variation of approaches are influenced by the lack of standardization, as well as the need to tailor methods to specific research objectives and experimental conditions [7]. The literature suggests that schizophrenia is often associated with abnormalities in gamma frequency, while bipolar disorder may be characterized by alterations in theta and delta frequency. In both disorders, the early detection of cognitive decline is critical, highlighting the potential significance of alpha frequency deviations. Moreover, MS analysis has been proposed as a potential marker. In schizophrenia, alterations in EEG microstates suggest cognitive and emotional dysregulation, with their association to gamma oscillations providing a potential diagnostic marker; in bipolar disorder, changes in microstate patterns are linked to mood states [11,12,21,36]. Cognitive deficits are typically more pronounced in schizophrenia, whereas mood instability is a key feature of bipolar disorder. In the latter, white matter involvement necessitates more advanced data collection and analysis techniques. The overlapping symptomatology of psychotic spectrum disorders and the asymptomatic nature of the premorbid phase require complex and sensitive tools capable of detecting subtle, early-stage changes, which are crucial for timely intervention and improved disease management [9,22,37,38,39].

## 3. Materials and Methods

### 3.1. Subjects

**Figure 1:**
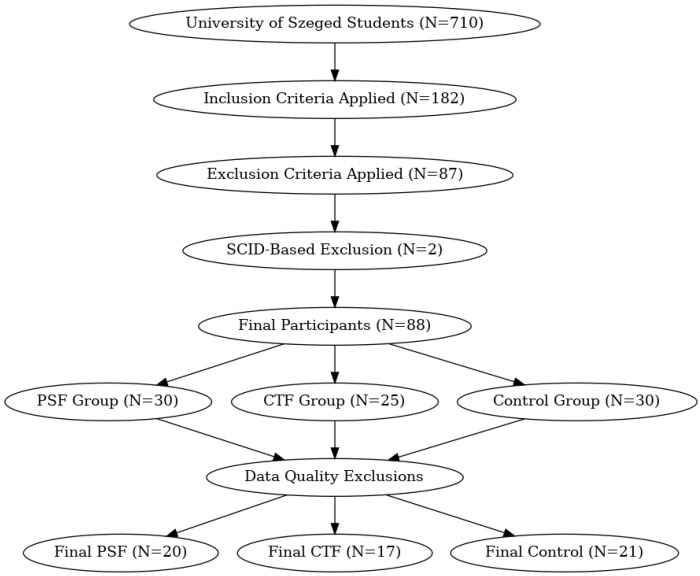
Flowchart of group formation.

Table 2 shows the selection criteria and Figure 1 illustrates the selection process. The investigation was conducted in conjunction with a broader scheme of thought entities: “An examination of neurobiological, cognitive, and neurophenomenological aspects of healthy volunteer students’ susceptibilities to mood swings or unusual experiences”. All the subjects gave written informed consent in accordance with the Declaration of Helsinki, and they were informed of their right to withdraw from the study at any time without providing any explanation. The selected participants received an expense allowance of HUF 15,000 for participation in the entire study, which was obtained through a grant application.

### 3.2. Assessments

The Temperament Evaluation of Memphis, Pisa, Paris, and San Diego Autoquestionnaire (TEMPS-A) [40] and the Hungarian version of the shortened Oxford-Liverpool Inventory of Feelings and Experiences (O-LIFE) were employed to select participants [41]. In addition, the Clinic Version of the Structured Clinical Interview for DSM-5 (SCID-5) [42] and Delusions Inventory (PDI) [43], the Mood Disorder Questionnaire (MDQ) [44], and demographic information (including age, sex, education, persistent illness not impacting neurocognitive functions, regularly prescribed medications, mental illnesses in the family, and previous psychiatric treatment, were also included.

The study employed several questionnaires, including the Examination of Anomalous Self-experiences (EASE) [45], Temperament and Character Inventory (TCI-R) [46], Morningness Eveningness Questionnaire (MEQ-SA) [47], The Behavioral Inhibition and Activation System Scales (BIS/BAS scale) [48], Leuven Affect and Pleasure Scale (LAPS) [49], Raven test [50], and THINC [51]. Additional instrumental assessments complemented these: eye-tracking antisaccade tests, instrumental self-aggression measures, actigraphy, and laboratory-based allostatic load evaluation.

### 3.3. Recording Procedure

The data were captured using the 32-channel BioSemi Active Two AD boxing ADC-12 instrument of the SZTE Institute of Psychology (channels: ‘Fp1’, ‘AF3’, ‘F7’, ‘F3’, ‘FC1’, ‘FC5’, ‘T7’, ‘C3’, ‘CP1’, ‘CP5’, ‘P7’, ‘P3’, ‘Pz’, ‘PO3’, ‘O1’, ‘Oz’, ‘O2’, ‘PO4’, ‘P4’, ‘P8’, ‘CP6’, ‘CP2’, ‘C4’, ‘T8’, ‘FC6’, ‘FC2’, ‘F4’, ‘F8’, ‘AF4’, ‘Fp2’, ‘Fz’, ‘Cz’). During the study, the subjects’ ‘resting-stage’ [15] brain waves were recorded in two states, one with closed eyes and the other with open eyes, in the rsEEG arrangement. In this study, we only examined the eyes-closed state EEG records.

### 3.4. Data Preprocessing

The data preparation and analysis required a methodical and thorough process to guarantee the integrity and usability of the EEG data. The dataset consisted of unprocessed EEG recordings, which underwent preprocessing using MNE-Python [52], a widely acknowledged library in neuroscience for analyzing electrophysiological data. The first step involved establishing a montage for the EEG data.

The following preprocessing steps were performed: first, the data were resampled from 8192 Hz to 128 Hz to improve processing efficiency. Next, a notch filter was applied at 50 Hz to eliminate power line noise. Then, a bandpass filter was used to isolate the frequency range of interest, specifically between 0.5 Hz and 60 Hz, while excluding high-frequency noise and slow drifts. EEG data cleaning required a hands-on approach, as confirmed by the manual intervention required to identify and interpolate faulty channels and intervals.

As data preprocessing is an integral component of microstate analysis, further details on this process can be found in the next section (Section 3.5.1), an external reference that provides in-depth descriptions of all the steps and techniques employed, with all examples of this GitHub repository: https://github.com/bilickiv/milabwp4/tree/akti_eeg_kodok/eeg, accessed on 6 February 2025.

### 3.5. Data Analysis

#### 3.5.1. Microstate Analysis

Microstate analysis in EEG involves identifying and analyzing transient, stable topographical patterns in brain electrical activity. These microstates provide insights into brain function by examining their temporal sequences. Preparing data for microstate analysis involves several critical steps: setting a montage, resampling, applying filters, artifact removal, and calculating microstate maps. Each step involves specific methods and parameters to ensure that the data are suitable for meaningful analysis. The analysis followed the steps described in Michel et al. (2018) [18]. After the preprocessing steps, artifact removal is performed by manually identifying bad intervals and channels. After data cleaning, some channels have bad data segments and they are interpolated to estimate their values based on surrounding signals if neighboring electrodes are functioning correctly at that segment. If interpolation is not possible due to widespread deviations, these segments are removed entirely. In some cases, entry channels were unusable. If many channels were corrupted, the EEG from the patient was deleted. Further artifact removal is performed using Independent Component Analysis (ICA) where necessary. After applying re-referencing, ICA helps in identifying and removing artifacts that are not easily correctable by simple filtering, such as eye blinks or muscle movements. This step ensures that the data predominantly reflect brain activity rather than external noise. Normalization is then performed to standardize the data. This involves scaling the signals to have consistent amplitude ranges across different recordings, which is crucial for accurate comparison and analysis. The segmented data are then clustered into distinct microstate classes. This involves using a modified K-means clustering to group similar EEG segments. The number of clusters, usually set to four, is predefined based on the expected variety of microstates. The algorithm iteratively assigns each segment to a cluster, minimizing the variance within each cluster. The resulting clusters represent different microstates, each characterized by a distinct topographical pattern. The analysis concentrates on the four most prevalent microstates, calculated at the peaks of the Global Field Power (GFP). GFP peaks are points in time where the spatial variance of the EEG signal is at its maximum, indicating the most pronounced topographical configurations. Those participants’ data that were unable to show the proper MS topological formation regardless of the rigorous data cleaning and preparation steps were discarded. This step was inevitable to ensure the data quality and the reliability of the final analysis. The clustered microstates are analyzed to understand their temporal properties, such as duration, occurrence, and transition probabilities. This involves calculating the average duration of each microstate, the probability of each transition from one state to another, and many more statistics and metrics. A flowchart illustrating the preprocessing steps can be found in Appendix A, Figure A5.

#### 3.5.2. Frequency Domain Analysis

EEGlib is a Python library specifically developed for feature extraction from preprocessed or raw EEG signals. EEGlib offers a comprehensive and standardized framework for the analysis of EEG data, facilitating the retrieval of an extensive array of features. By employing the default settings on our 32-channel, 128 Hz preprocessed EEG data, we conducted every analysis at one-second intervals, thereby generating a secondary time series. The dimensions of the outputs generated by this method are not optimal for machine learning. To resolve this issue, four summary statistics—the mean, standard deviation, upper quartile, and lower quartile—are computed for each time series during the feature engineering phase. By capturing the distribution of the time series, these metrics guarantee the preservation of the most informative elements, which are then applicable to machine-learning models. Consequently, a specific attribute is denoted by four numerical values appended to its name: “_mean”, “_std”, “_upper_qrt”, and “_lower_qrt” (“_stat”). A feature name consists of the following components: the sign of the implied statistic, the name of the channel (“channel”), the frequency band (“_frequency”), if present, and the name of the metric (“_stat”). Table A1 lists all the features and their corresponding names, which are described in Appendix B.3. Due to the wide variation in scale, we first implemented a standard normalization. Despite this, the scikit-learn Python library faced difficulties when dealing with values that were extremely close to zero, and treated them as zero. To tackle this issue, we implemented a logarithmic transformation on features that had a standard deviation below 10^−4^ and subsequently applied standard normalization.

### 3.6. Machine Learning

In this section, we intend to introduce the novel ML-based feature selection and analysis tools we employed in our research. Since those methods resemble some unique perspectives, we aim to provide a comprehensive overview.

#### 3.6.1. AHFS

**Figure 2:**
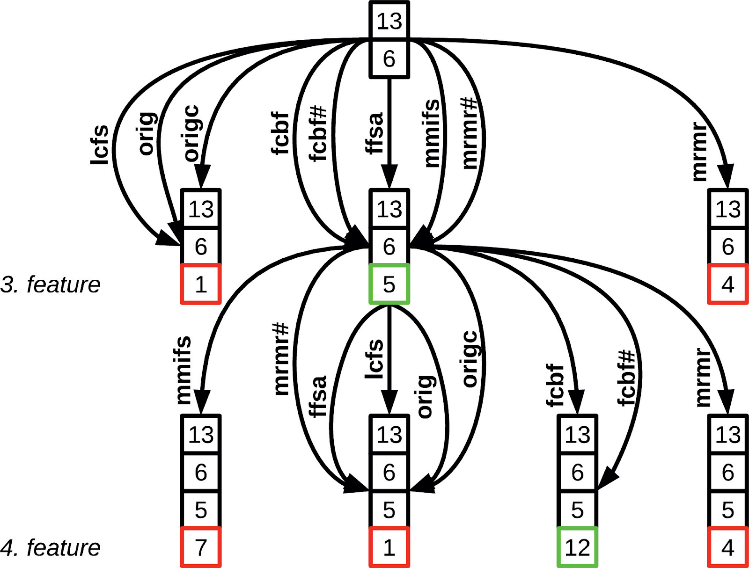
The AHFS algorithm is visualized through an operational graph, delineating two consecutive steps. The functionality of the proposed algorithm is demonstrated using the Housing dataset by Lichman et al. (2013) [53]. The nodes within the graph are annotated with feature indices represented as numerical values and enclosed in frames of diverse colors. These color distinctions denote the status of the analyzed variables. Specifically, features with black frames signify the previously selected feature set in the current state, while features with colored frames (red, green) are considered candidate features within the set. The green-framed feature denotes the optimal choice, exhibiting the smallest estimation error or the highest accuracy when compared with other potential variables enclosed in red frames. The figure originated from Viharos et al. (2021) [23].

The Adaptive, Hybrid Feature Selection (AHFS) algorithm offers an innovative approach to feature selection in machine learning [23]. It combines supervised feature selection techniques, each with specific evaluation measures, to create a versatile solution. Utilizing correlation and information-theoretic-based measures such as MMIFS, mRMR, LCFS, and JMIM, among others, the algorithm evaluates the relationship between features and their information content. This hybrid approach allows for integrating additional feature selection methods and metrics, ensuring adaptability to various scenarios and future research findings. For feature selection, the AHFS algorithm (Figure 2) employs the widely adopted Sequential Forward Selection (SFS) technique.

This technique incrementally expands the selected feature set by adding one feature during each iteration based on their contribution to minimizing the estimation error or maximizing accuracy. Consequently, adaptivity is a fundamental aspect of the AHFS algorithm, as it explores not only within the feature space but also in the realm of feature selection techniques and evaluation measures simultaneously. This adaptivity enables the algorithm to assess different feature selection methods at each step, offering a comprehensive and effective approach. Its robustness and effectiveness in feature selection position the algorithm as suitable for real-world applications, including the screening of mental health conditions.

AHFS was executed on all calculated features. A single “run” of the algorithm begins by selecting an initial feature, then proceeds up to 20 iterations, each time adding one new feature and training a corresponding model (as illustrated in Figure 2). Because there is some randomness in the choice of the “best” feature at each step—particularly in later iterations—20 runs were performed to ensure feature stability. AHFS uses 3-fold cross-validation and provides performance metrics averaging the 3 training sessions. The top 20 models had Shapely calculations to ensure comparable results with CFFS.

#### 3.6.2. Clique Forming Feature Selection

The Clique Forming Feature Selection (CFFS) method, initially introduced by Nagy et al. (2023) [24], was further enhanced and elaborated upon in this study. Similar to AHFS, this approach also integrates machine-learning algorithms to select features, but in such a way that the widest spectra of the available features have a chance to “compete” for a better prediction. This approach is important in medical research, to balance model performance with understanding the underlying mechanisms. In this investigation, we utilized Logistic Regression (LR), Random Forest (RF), and one simple Artificial Neural Network (ANN), which are discussed in detail in Appendix B.1.

**Figure 3:**
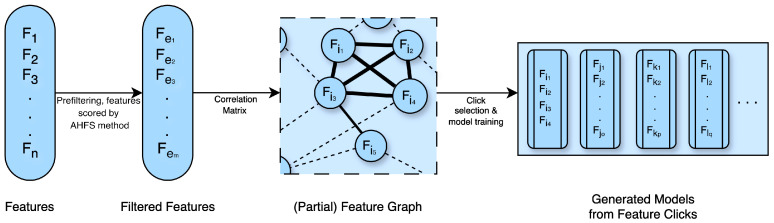
Workflow of the Clique Forming Feature Selection (CFFS) algorithm. The process begins with the full feature set ($F_{1},F_{2},F_{3},\ldots ,F_{n}$) ($F_{e_{1}-e_{m}}$). The filtered features are used to construct a weighted feature graph, where edges represent pairwise Pearson correlations. For example, $F_{i_{1}-i_{4}}$ is a 4-element whole subgraph (clique) and will be a feature candidate; however, $F_{e_{5}}$ was left out from the analysis because it does not have enough low correlation features. Edges with absolute weights above a threshold are removed, resulting in a general graph. Cliques ($F_{c1},F_{c2},\ldots ,F_{ck}$), defined as fully connected subgraphs, are identified as potential feature sets. If too many cliques result, a random selection is made. These feature sets are used to train models with three machine-learning algorithms. The models are evaluated using 3-fold cross-validation. Shapley values are computed for feature importance, and aggregated Shapley tables provide robust rankings for the selected features.

Figure 3 illustrates the steps of the CFFS algorithm and outlines a comprehensive pipeline for feature selection and model training. This approach makes it possible to minimize inter-correlation in feature set candidates.

**Figure 4:**
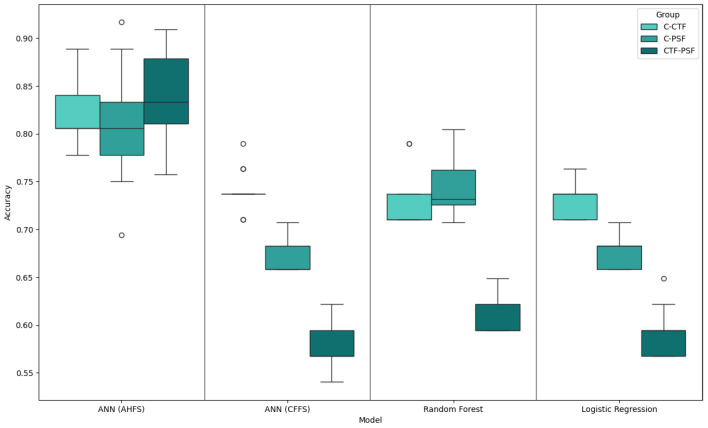
Accuracy box plot of the top-performing models. The labels show which two groups were separated.

Due to the enormous number of features, we prefiltered them based on the AHFS findings. Those features are selected, which appear at least once in the 20 runs, ending up with 35 features in both cases. Applying 0.4 (PSF) and 0.35 (CTF) thresholds to form common graphs, approximately 5000 cliques are identified, and from these, 600 sets of varying sizes (3–9 features) are randomly selected, ensuring broad coverage of the overall feature space. These feature sets are used to train models with three machine-learning algorithms (LR, RF, ANN). The models are evaluated using 3-fold cross-validation, and the top 20 models per algorithm are selected based on accuracy. These accuracy values are presented in the next section (see Figure 4).

Subsequently, we aggregate the Shapley values from these top-performing models, which makes for a more reliable interpretation by increasing feature stability (truly globally important features are selected) and filtering out inconsistent predictions. Also, due to the small inter-correlation, a huge drawback of Shapley value calculation is canceled out; namely, this feature importance calculation method tends to distribute importance among correlated features. Reducing the inter-correlation among feature sets leads to more reliable results. Applying three different ML algorithms ensures that all kinds of underlying connections are observed. LR tends to find linear connections, and RF is much more sensitive to finding non-linear relations; on the other hand, ANN could pick up much more complex structures. Those solutions make CFFS a unique method designed especially for medical and similar data analyses, where not only model performance but also the investigation of underlying mechanisms is paramount. Since we used cross-validation, each fold’s predictions and Shapely values were saved and processed equally, so all data points contributed to the analysis. Further details of the method are described in Appendix B.2.

**Figure 5:**
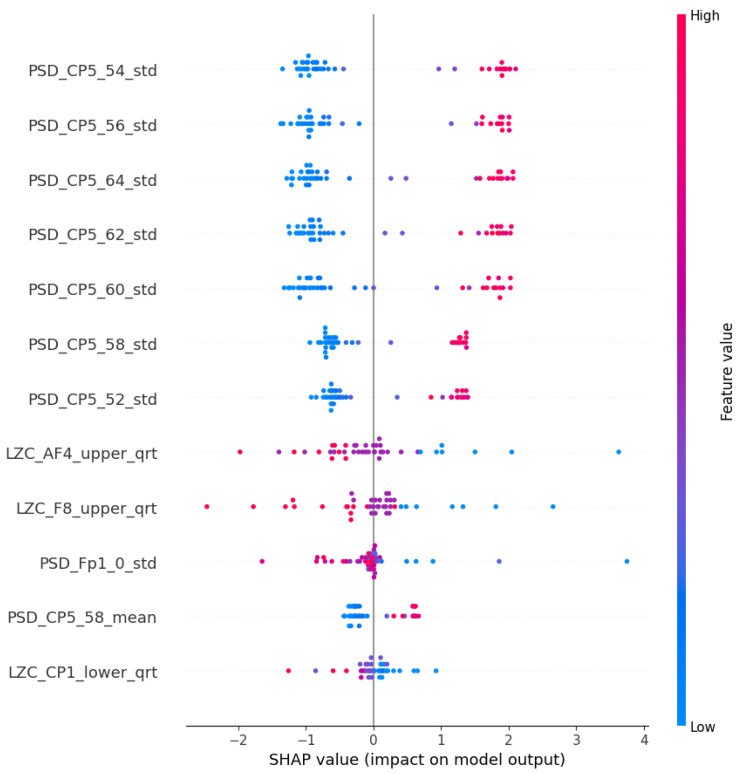
The SHAP summary plot of the LR models in the PSF group. The top 12 features were ranked by the importance score.

With this procedure, we were able to select and evaluate models that exhibited satisfactory accuracy levels, while utilizing the Shapley values to gain insights into the contributions of different features toward the model predictions. This approach offers a more comprehensive analysis of the group and helps provide a reliable framework for further investigations. The aggregated Shapley values of the features are displayed in the SHAP “Summary plot” (for example, see Figure 5), which depicts the aggregated Shapley values. In each row (a particular feature), each point corresponds to a decision on a particular participant. The x-axis position of the point represents its impact on the model (in this context, the overall impact across all different, aggregated models) output, colored by the particular feature’s relative value (high or low). Points further left indicate a higher contribution to labeling as a risk factor, while points further right indicate a higher contribution to labeling as control. The other types of plot are the comparative ones, where the mean of the absolute values of the Shapley values (importance scores) are normalized. This normalization ensures that the importance score adds up to one.

## 4. Results

### 4.1. Generated Models

The method used is based on training several simpler models and explaining them one-by-one, then aggregating to obtain the importance scores; therefore, we represent our results with box plots, shown in Figure 4. The analysis constructed from three binary classifications, two cases where the control group gets separated from the PSD (C-PSF), and then the CTF group (C-CTF), and a third case, where the two latent groups get separated from each other (CTF-PSF). Since we utilized cross-validation, each fold, and consequently each sample, was presented in both the training- and testing-datasets. Because of this, each sample obtained and saved the prediction; therefore, the presented accuracy is composed by using all data points. The best accuracy was achieved using the AHFS method (Table 3). Owing to the use of different running environments and optimization procedures, the outputs cannot be compared unambiguously, but the AHFS was able to separate PSF and CTF groups, so we can move forward, relying on these models in the following.

### 4.2. PSF Group Findings

**Figure 6:**
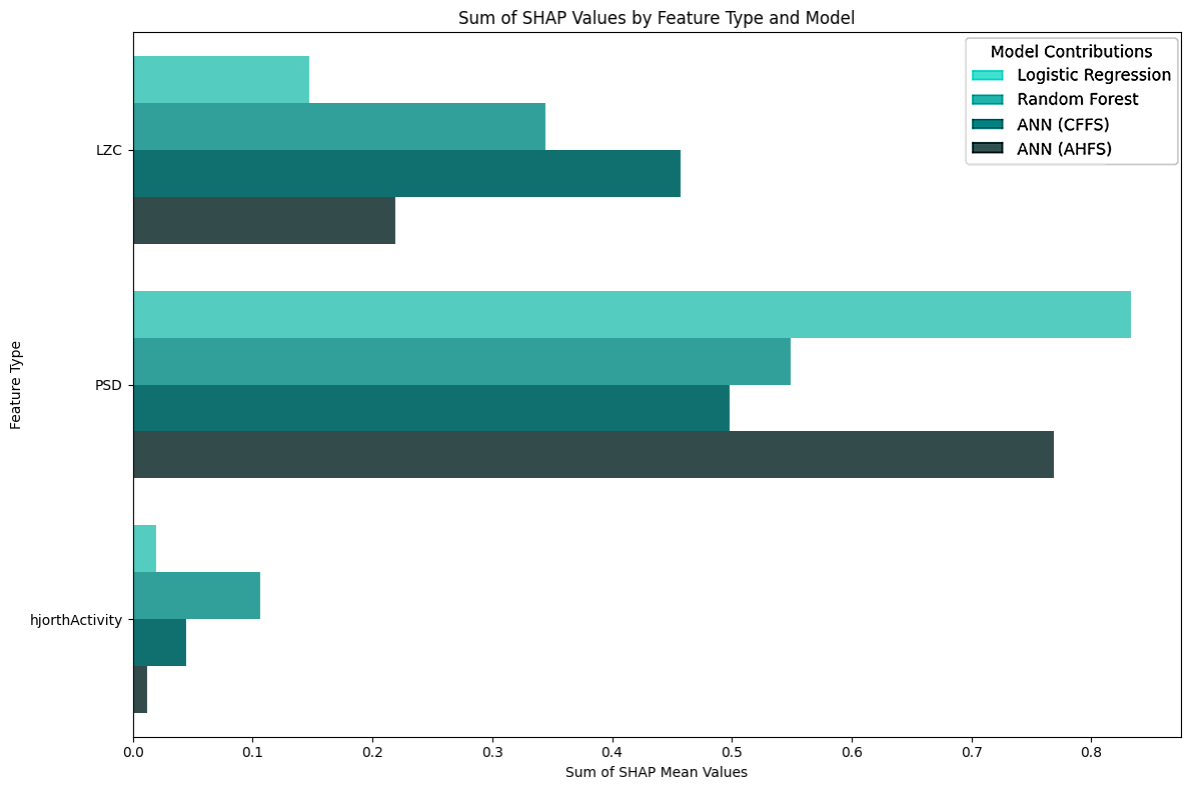
Separation of importance score ratios by feature type in the PSF group. The PSD row shows the frequency-dependent score ratio. The algorithms are differentiated by the colors. The PSD-related are the most important feature types, followed by the Lempel-Ziv complexity-related features.

**Figure 7:**
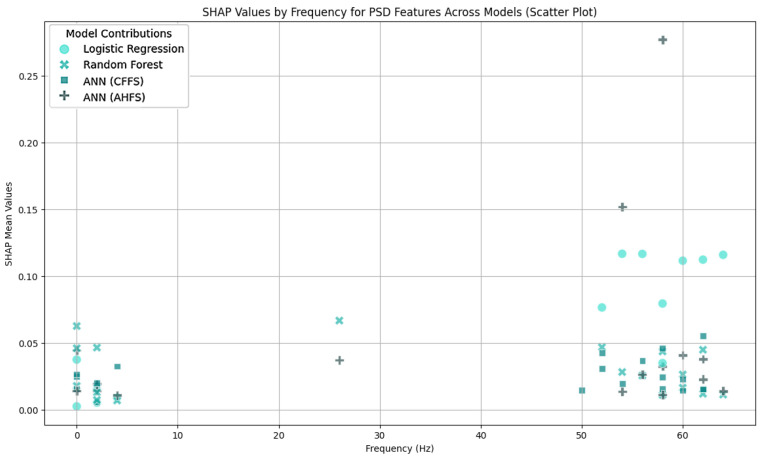
A scatter plot as a function of frequency range and importance scores in the PSF group. The importance scores are clustered under 5 Hz and above 50 Hz. The algorithms are differentiated by colors.

**Figure 8:**
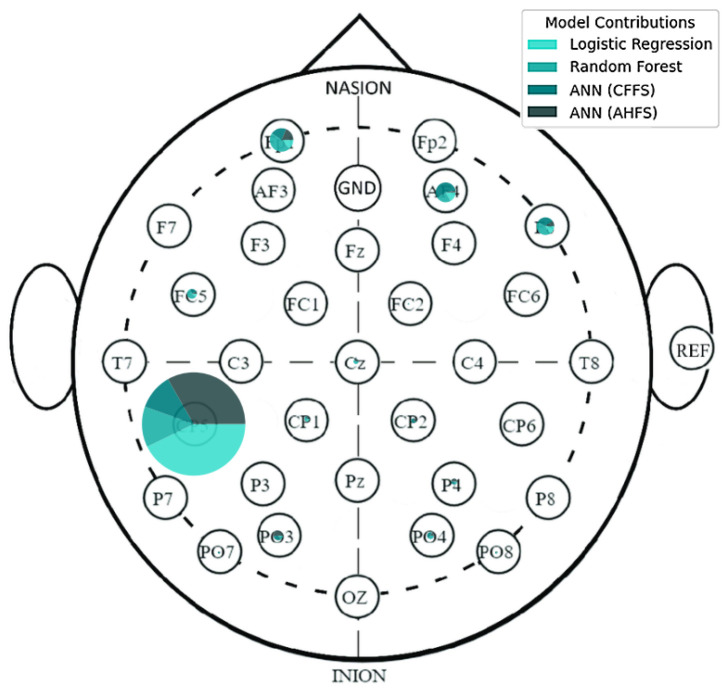
A spatial distribution of the relative importance scores in the PSF group, displayed on the electrode scalp diagram. The majority of the importance scores are located at the CP5 electrode, and some others on the frontal lobe. The algorithms are differentiated by colors.

**Figure A1:**
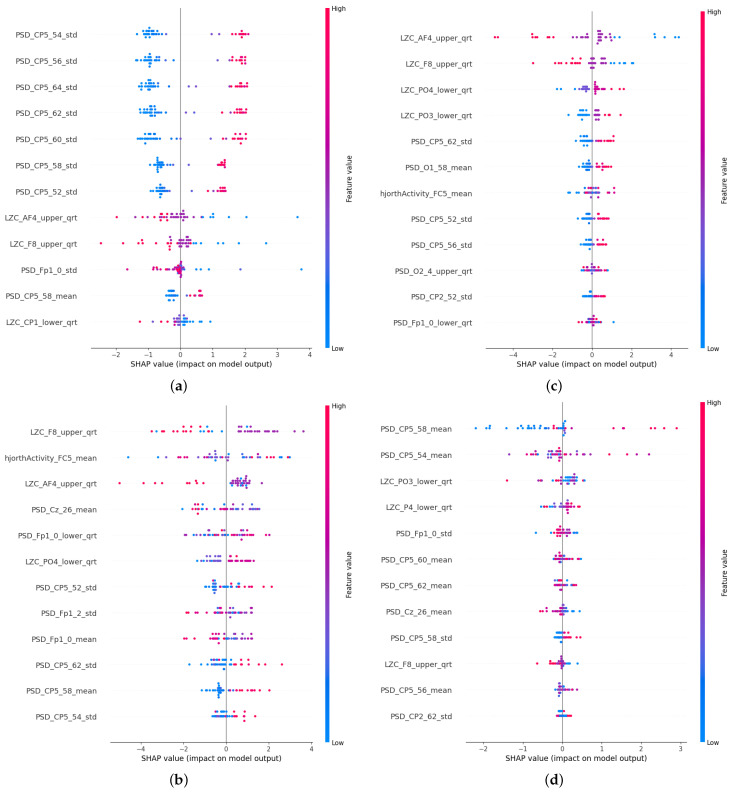
(**a**) Summary plot of the Logistic Regression models’ Shapley values of the PSF. (**b**) Summary plot of the Random Forest models’ Shapley values of the PSF. (**c**) Summary plot of the ANN (CFFS) models’ Shapley values of the PSF. (**d**) Summary plot of the ANN (AHFS) models’ Shapley values of the PSF.

The LR algorithm is shown in Figure 5, while the other three algorithms are listed in Appendix A, Figure A1b–d. The most interesting results are the first seven features of LR. The standard deviation of PSD across the frequency range of 50–60 Hz at the CP5 electrode is substantially larger. A similar result was noted in the ANN (AHFS) (Figure A1d), but the mean of the PSD features were affected. Using Figure A3a, Appendix A, it is straightforward to compare the relative importance scores of the features and also to see that certain features appear in the other groups, but with different significance values. Figure 6, Figure 7 and Figure 8 show the importance scores from various aspects, and it confirms that most of the algorithms used the features of the channel CP5 in the low (<7 Hz) or in the high (50–65 Hz) frequency range.

### 4.3. CTF Group Findings

**Figure 9:**
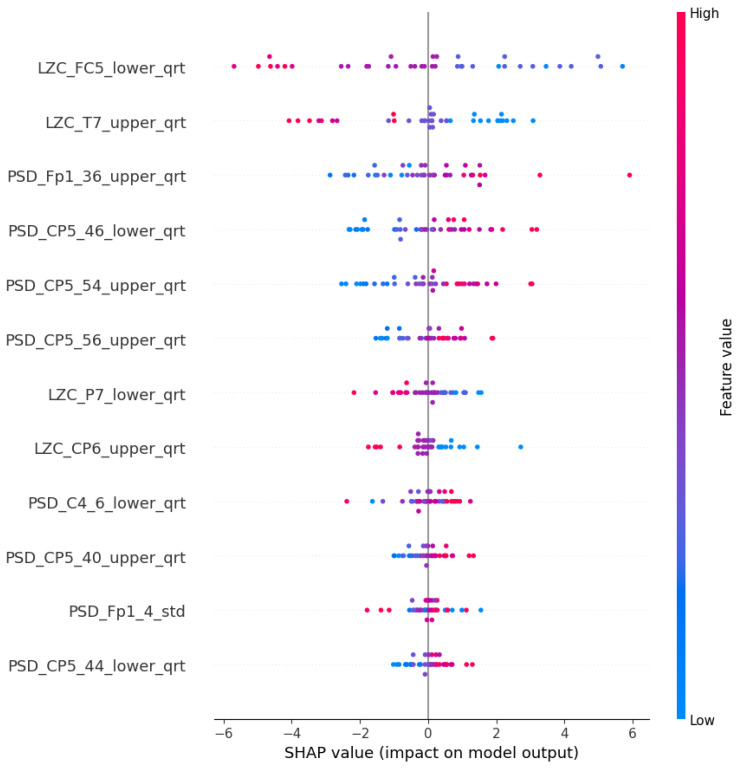
The SHAP summary plot of the LR models in the CTF group. The top 12 features are ranked in order of importance scores.

**Figure 10:**
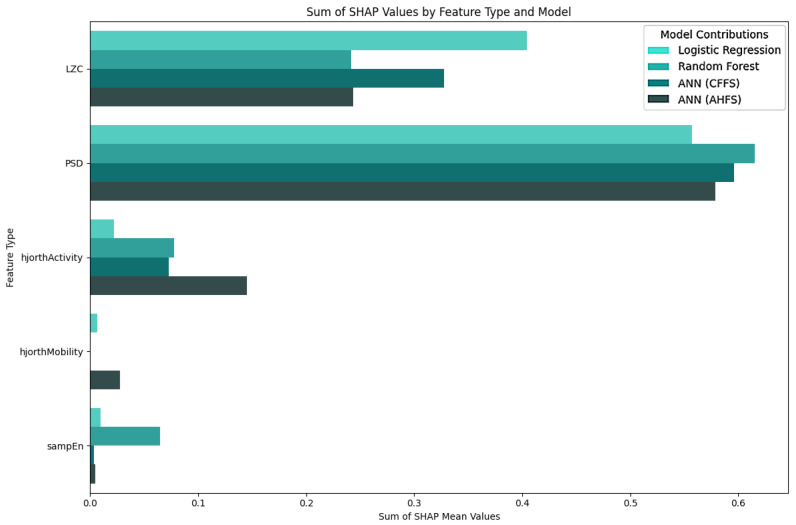
Separation of importance score ratios by feature type in the CTF group. The PSD row shows the frequency-dependent score ratio. The algorithms are differentiated by colors.

**Figure 11:**
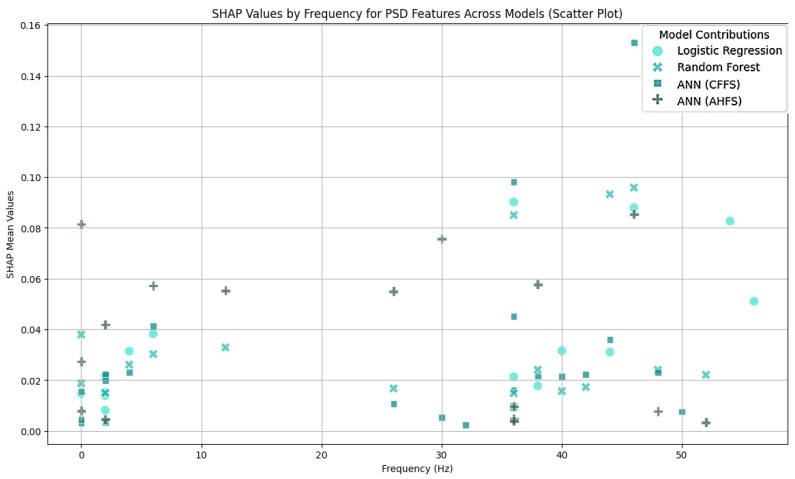
A scatter plot as a function of frequency range and importance score in the CTF group. The algorithms are differentiated by colors.

**Figure 12:**
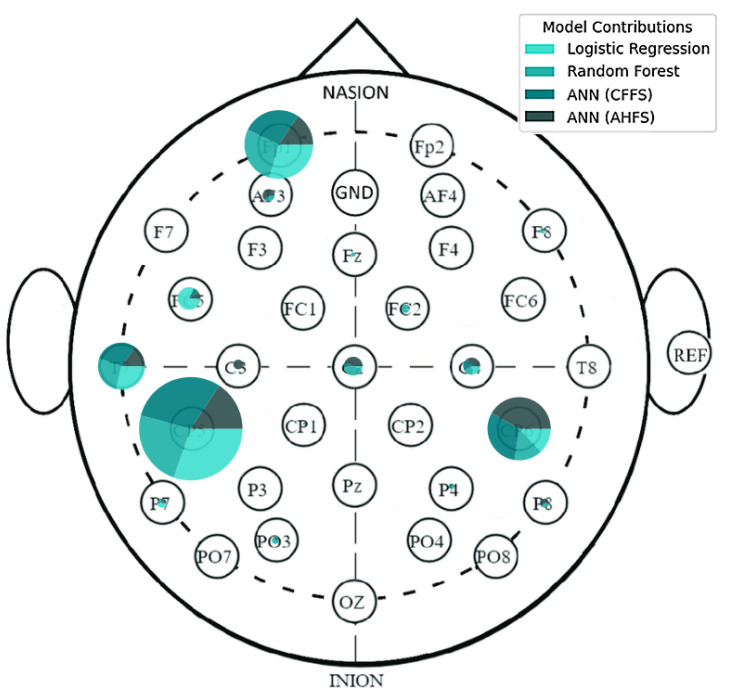
A spatial distribution of the relative importance scores, displayed on the electrode scalp diagram in the CTF group. The colors differentiate the algorithms.

**Figure A2:**
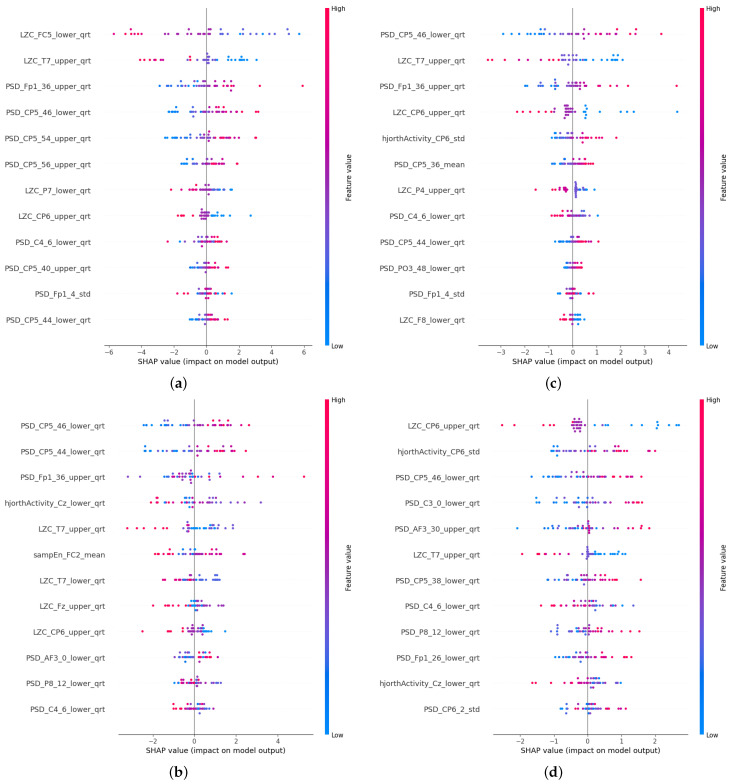
(**a**) Summary plot of the Logistic Regression models’ Shapley values of the CTF. (**b**) Summary plot of the Random Forest models’ Shapley values of the CTF. (**c**) Summary plot of the ANN (CFFS) models’ Shapley values of the CTF. (**d**) Summary plot of the ANN (AHFS) models’ Shapley values of the CTF.

**Figure A3:**
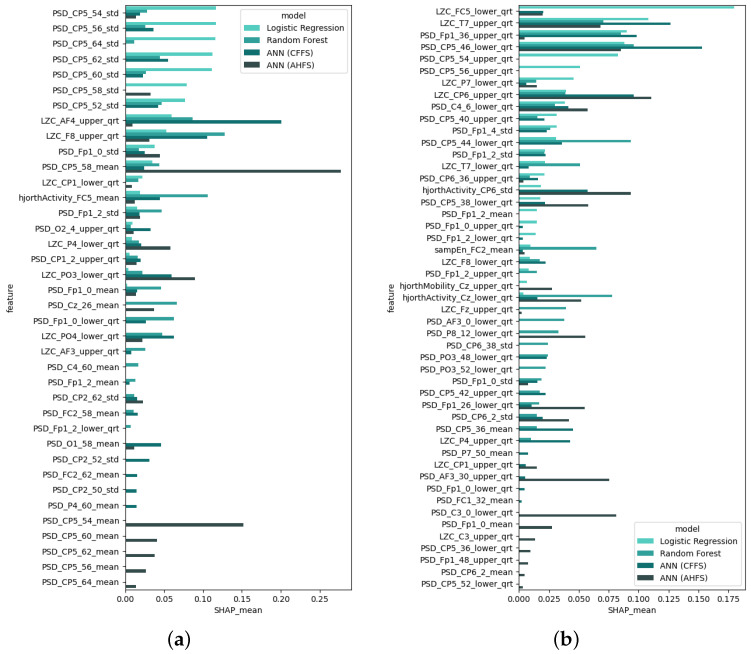
The values from the four algorithms distinguished with colors. (**a**) Relative importance scores of PSF group. (**b**) Relative importance scores of CTF group.

The Shapley values of the features judged important by the different algorithms are shown in Figure 9 and Figure A2b–d in Appendix A. The relative importance scores for all the features are shown in Appendix A, Figure A3b. The feature type (Figure 10), frequency (Figure 11), and spacial distribution (Figure 12) are shown below. For LR, the first two ranked features are the Lempel-Ziv complexity measure, for electrodes FC5 and T7, both with reduced complexity for the susceptibility group. This algorithm used the FC5 electrode by itself, while the other three models found this channel to be of little importance in any form. “LZC_T7_upper_qrt” is also ranked second to the RF algorithm, with a similar distribution, and a little bit lower ranked to the two ANN algorithms (Figure A2b–d). RF produced balanced, well-distributed values. RF and ANN (CFFS) found the same feature to be the most important; for the PSD of the 44–46 Hz frequency range of the CP5 electrode, these values were elevated in the susceptible group. Similarly, LR feature 4, 5, and 6 showed an increase in features (on CP5) in the lower range of the gamma for the observed group, and similar results were observable for RF. Thirdly, the PSD of the 34–36 Hz frequency range of Fp1 was the same for the three algorithms, with an increase in susceptibility. Almost all features include “_upper_qrt” or “_lower_qrt” features. This tells us that there are differences in the lower or upper quartile of the distributions of the scores counted on a person’s data for control and bipolar predisposition. Hence, it is more likely to be the sparser values that appear in the scores that are different, rather than the whole distribution being shifted or even spread out differently. These results also help us to better understand our groups.

### 4.4. CTF-PSF Comparison Findings

**Figure 13:**
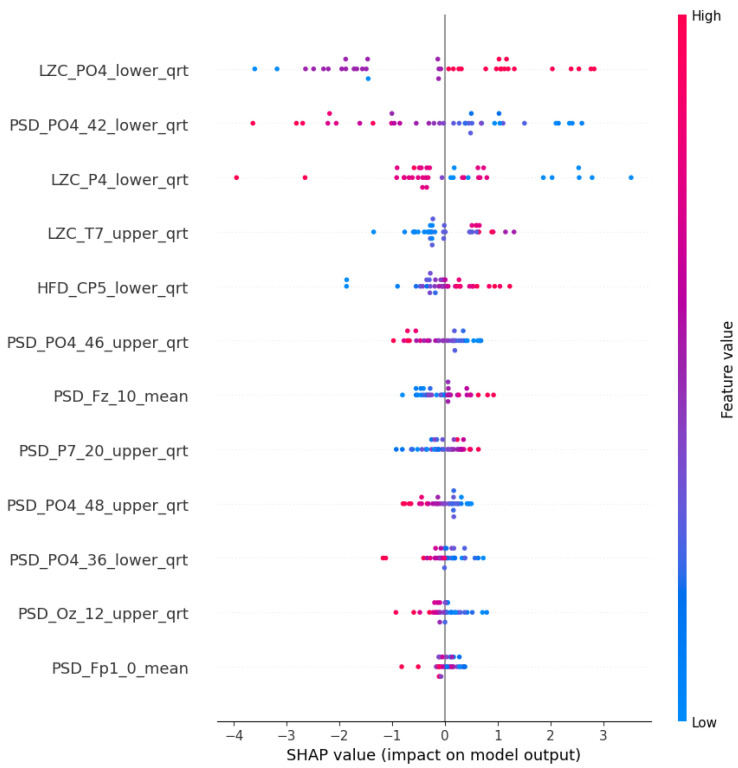
The SHAP summary plot of the ANN (AHFS) models’ top 12 features in CTF and PSF group comparison.

**Figure 14:**
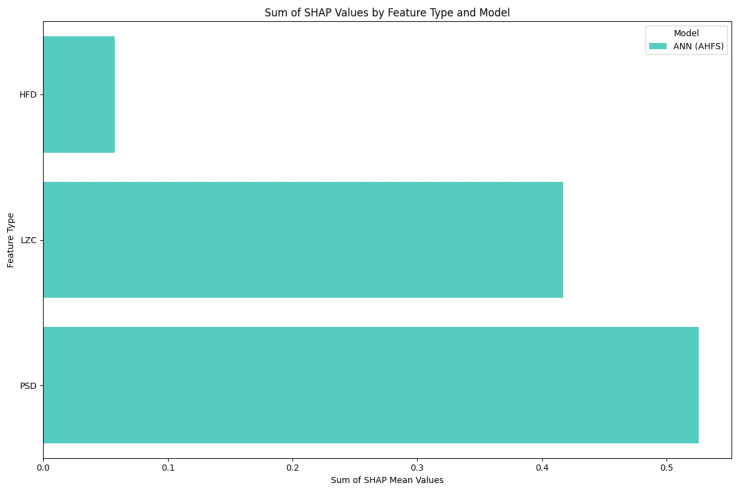
Separation of importance score ratios by feature type in PSF and CTF group comparison. The PSD row shows the frequency-dependent score ratio. The algorithms are differentiated by colors.

**Figure 15:**
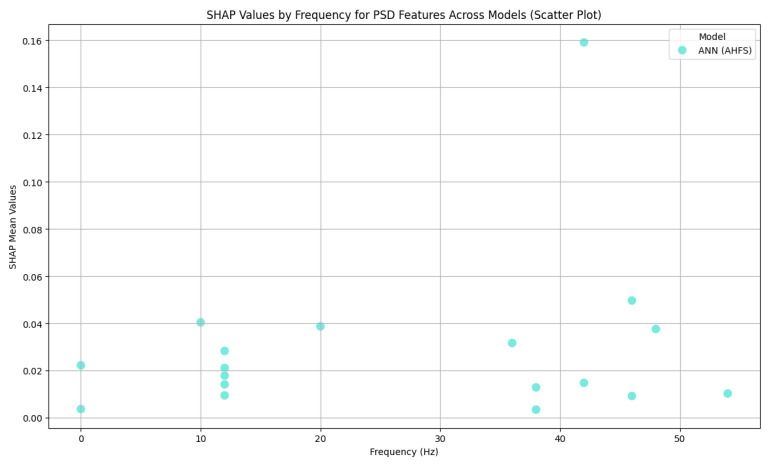
A scatter plot as a function of frequency range and importance score in CTF and PSF group comparison. The algorithms are differentiated by colors.

**Figure 16:**
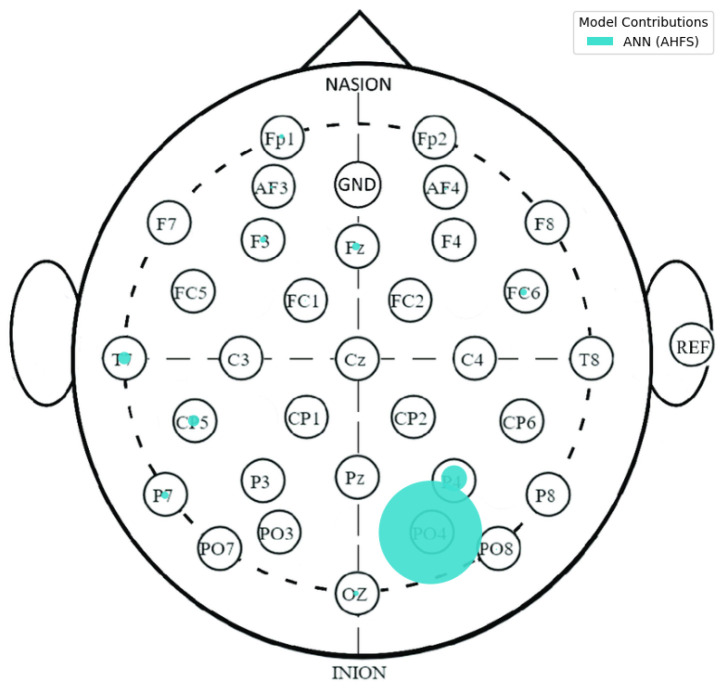
A spatial distribution of the relative importance scores, displayed on the electrode scalp diagram in CTF and PSF group comparison. The algorithms are differentiated by colors.

**Figure A4:**
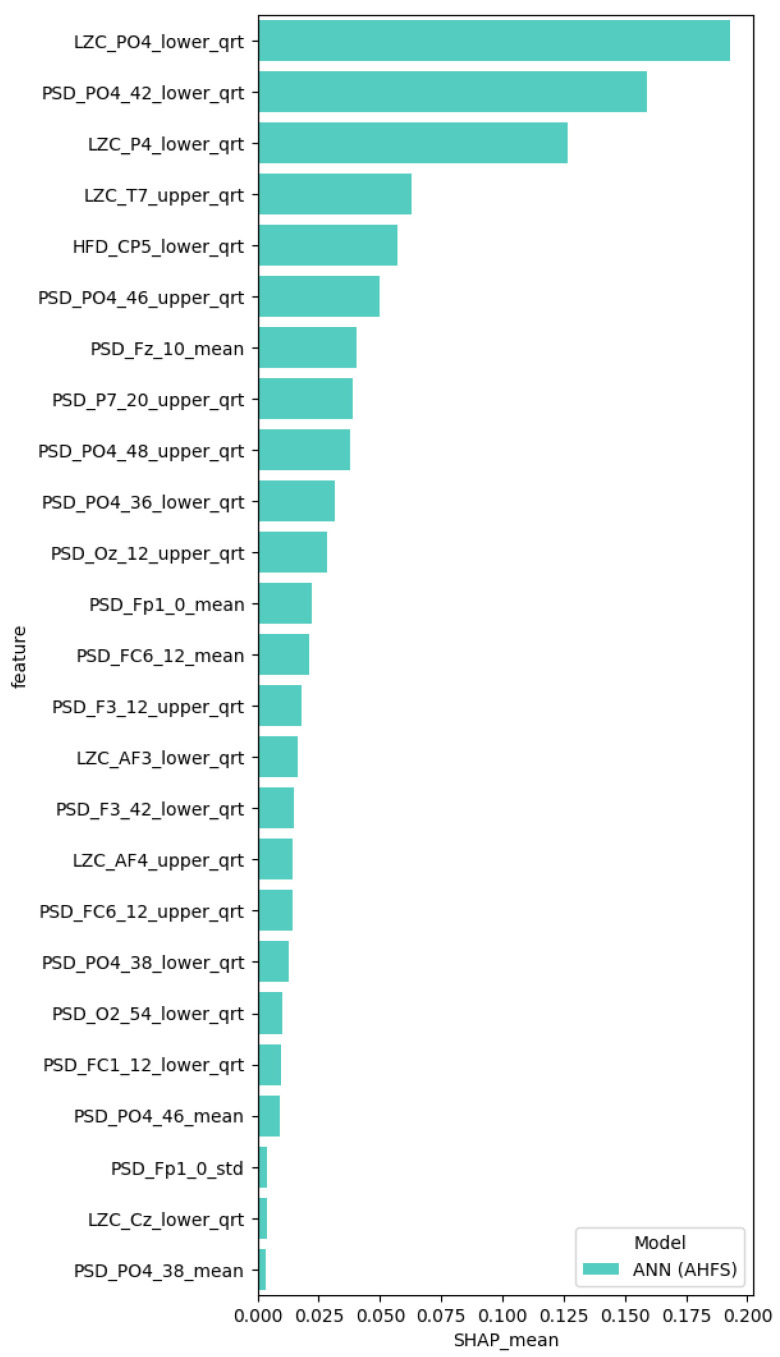
Relative importance scores of the PSF and CTF group separation, displaying only the ANN (AHFS) models.

**Figure A5:**
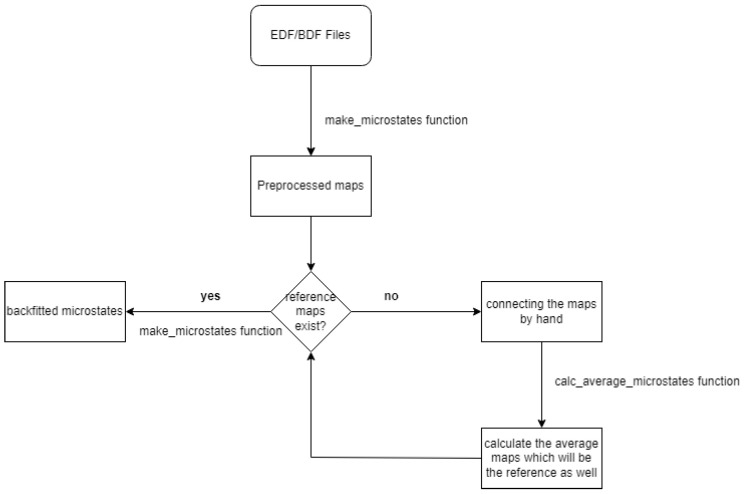
Preprocessing steps and mapping of each step’s utilized program code. The program codes are contained in the GitHub repository.

Figure 4 indicates that none of the models, except the AHFS (ANN), performed well in separating the two propensity groups, so only the results of using this method are discussed here. Since the algorithms here do not predict the presence of susceptibility, positive Shapley values do not indicate the presence but the susceptibility of schizotypy, while negative ones indicate latent bipolarity (Figure 13). The whole feature set and their importance scores are listed in Appendix A, Figure A4. Figure 14 display the types of important features, showing the majority PSD and LZC features, while Figure 15 show the important PSD frequencies. The electrode scale diagram (Figure 16) also clearly shows that the PO4 channel played a key role. PSD features in this channel covered the frequency ranges 36, 42, 46, and 48 Hz. The quartiles are shifted, so a slight separation in the distributions occurred in the same feature groups, and a slight decrease in the PSF compared to the CTF group was seen. Figure 15 reveals that the overall importance score distribution across the frequency ranges was more spread. Also, there is a marked increase in LZC complexity in favor of the PSF group on the PO4 channel. Overall, the other channels are not significant, but it is interesting to note that the third feature (channel P4) displays an increase in LZC complexity in the CTF group, in contrast to, and again for PO4 on T7.

## 5. Discussion

Here, we sought to differentiate between groups of healthy university students basedon their latent liability of developing schizotypy or bipolar disorder using resting-state EEG data. Utilizing machine-learning methods capable of delivering effective results even with small samples, we attempted to segregate those with low risk from their peers who do not exhibit these latent vulnerabilities, as well as from each other. The characteristics generated by the sophisticated artificial-intelligence models provided the means to effectively distinguish the risk groups from the control group and, to a certain extent, from each other. The models generated exhibited a number of important features based on the algorithms employed. However, the novelty of the present research makes it challenging to ascertain their precise role. In most cases, these results can be compared with those of participants at high risk or with with manifested disease conditions [25].

### 5.1. Frequency Bands

The majority of the features identified as crucial in the PSF group by the models derived from the frequency analysis (features names start with “PSD” (Power Spectral Density)) were quantified within the gamma range. This indicates that the variation in gamma frequency presence between groups was important according to the algorithms. The majority of these features were associated with the CP5 channel (13 features), which was also identified as the most significant channel in the model. Also, the following channels were included: CP2 (three features), FC2 (two features), C4 (one feature), P4 (one feature), and O1 (one feature). The majority of features in the CTF group were also measured in the gamma range. Once more, the majority of these features are associated with the CP5 channel (10 features), which is the most significant electrode in this group according to the models. Furthermore, the gamma range is present for channels Fp1 (2 features), CP6 (2 features), PO3 (2 features), P7 (1 feature), AF3 (1 feature), and FC1 (1 feature). It can be seen that the features in the PSF group are generated from frequencies between 52 and 64 Hz, while for the CTF group the range is between 30 and 56 Hz (considering all the selected features based on Figure A3, not only the most significant ones highlighted in Section 4). The importance of gamma frequency for psychosis spectrum disorders is discussed in Reilly et al. (2018) [36], which provides a synthesis of previous literature on the topic. The study enumerates several factors that must be considered to ascertain the significance of the frequency range. However, the principal conclusion is that it is hard to determine in the early stages of psychosis spectrum disorders. Consequently, it is difficult to draw a precise conclusion from the literature based on the present results. Other noteworthy frequency bands included delta, theta, alpha, and beta. It should also be mentioned that the frequency analysis literature is inconsistent. However, it does highlight some possible guidelines along which the issue is worth investigating [11,14]. In the PSF group, the delta wave was important for the Fp1 channel (6 features) and the beta wave for the Cz channel (1 feature). The extant literature suggests that deviation of the delta wave at frontal regions may indicate the presence of reduced negative symptoms in first-episode schizophrenic patients, with no reports for the premorbid phase, so even with the importance of the frontal channels (FC5, Fp1, F8) in the model, it is hard to reach a definitive conclusion for our results [16]. In the CTF group, the delta wave was important for channel Fp1 (seven features), channel CP6 (two features), channel AF3 (one feature), channel C3 (one feature), the theta wave for channel Fp1 (one feature), channel C4 (one feature), and the alpha wave for channel P8 (one feature). It is noteworthy that the literature does not provide details regarding the localization of these differences. Consequently, the exact interpretability of these findings for the premorbid phase remains to be elucidated [14,21].

### 5.2. Complexity

Another component of the feature set was the Lempel-Ziv complexity. Complexity testing, adapted from computer science, is a common practice when examining data generated by brain imaging in psychiatric disorders. This is due to its ability to measure complex and dynamic events, which makes it suitable for application to EEG data in schizophrenia and bipolar disorder [54,55,56]. The extant literature indicates that the results are inconsistent in schizophrenia. Increased as well as decreased complexity is variably reported, which may depend on the duration and phase of the disease as well as the affected area [57,58,59]. A paucity of literature is available on bipolar disorder, with the available results suggesting abnormally elevated complexity in the medial temporal gyrus and the medial frontal gyrus as potential markers. This may be associated with symptoms of the disorder and cognitive functioning. In the CTF group, the first and second features of the FC5 and T7 electrodes were derived from Lempel-Ziv complexity. The results did not demonstrate a clear direction along the axis of elevated and reduced complexity. The significance of this result is difficult to determine from the literature [59].

### 5.3. Additional Features

Additional features included hjorthActivity, hjorthMobility, and SampEn. In the PSF group, the feature hjorthActivity_FC5_mean was selected for the models, while in the CTF group the features hjorthActivity_CP6_std, hjorthActivity_Cz_lower, hjorthMobility_Cz_upper, and sampEn_FC2_mean were selected for the models. The majority of existing literature on these statistical variables relates to schizophrenia, with no relevant studies identified for bipolar disorder [60,61,62]. The available evidence suggests that the use of these indicators of complexity may offer a promising avenue for further research. It is not easy to ascertain the importance of the features derived from these indicators in the context of the present study, as well as the relevance of including one feature from the “hjorth” category and three from the “sampEn” category in the PSF group and one from the “hjorth” category in the CTF group in one of the models.

### 5.4. Localization of the Features

The examination of schizophrenia and bipolar disorder is challenging due to their varying and overlapping symptoms and the lack of a standardized approach in research. A more comprehensive approach, considering multiple biomarkers and levels of neurological alterations, is needed to better understand these disorders [63,64]. The discussion focuses on factually describing the features generated by algorithms and the brain areas associated with these features in both schizophrenia and bipolar disorder. It is important to note that brain areas often have overlapping functions, so multiple electrodes may be associated with the same brain area and be presented in groupings [65,66,67].

### 5.5. Features of the PSF Group

Figure A3a shows the features in the PSF group that are jointly considered important by the different models. Figure 6 shows the feature type, Figure 7 the frequency, and Figure 8 the location distribution of the important scores, and Figure A1 in Appendix A shows the SHAP summary plots of all four algorithms. Table 4 summarizes the relevant electrodes and the associated brain areas.

The areas BA39, BA10, BA9, and BA7 (as electrodes CP5, Fp1, AF4, AF3, CP1, CP2, P4) are responsible for cognitive and executive functions. CP5 (L. BA39) is a key area within this grouping, as it is responsible for the localization of the majority of features. Furthermore, it is associated with features of higher importance in the models, in addition to features of lower importance in the models. The area is responsible for a number of functions, including face recognition, reading (text detection), and visuomotor orientation [65]. The areas BA45, BA6, BA4, and BA1 (F8, FC5, FC2, Cz, C4) are responsible for motor activation and somatosensory functions. BA19 and BA18 (PO3, PO4, O2, O1) constitute part of the visual cortex and are responsible for the recognition of movement, colors, faces, and objects [65]. It is well established that disturbances in most of these functions are readily detected and measured in individuals with established schizophrenia (e.g., cognitive and executive functions, motor activation, social cognition). In the premorbid phase, the principal symptoms observed include delayed motor development, attentional dysfunction, language comprehension dysfunction, poor academic performance, social isolation, and disturbance of emotional functioning. Also, findings are inconclusive regarding the involvement of problems with processing speed, verbal learning and memory, executive functions, and social cognition disorders, although some of the features (e.g., features of FC5 and Fp1 electrodes) fall within areas that may include these problems [39,68]. Potential disturbance of functions of the regions encompassed by the electrodes deemed important by the models are analogous to the symptoms of schizophrenia. Given schizophrenia’s impact on neurodevelopment [63], it is possible that non-detectable underlying abnormalities might already exist. Furthermore, since our methodology successfully distinguished the research group from the control group, our findings suggest that current methods in this research area may be insufficient for detecting and identifying premorbid abnormalities with sufficient precision. Further research is necessary to confirm our results and to identify specific brain functions, areas, or networks that might be crucial for early disease detection.

### 5.6. Features of the CTF Group

Figure A3b shows all the features in the CTF group that were found to be important by the different algorithms. Figure 10 shows the feature type, Figure 11 the frequency, and Figure 12 the location distribution of the importance scores, and Figure A2 from Appendix A shows the SHAP summary plots generated by the four algorithms. Table 5 summarizes the relevant electrodes and the associated brain areas.

The BA45, BA6, BA4, and BA1 (F8, FC2, FC5, Fz, FC1, Cz, C3, C4) areas are involved in motor development. These areas also had importance in the PSF group, with a distinction being the higher significance attributed to the left BA6 (FC5) area in the CTF group compared to the PSF group. The most prominent feature is at the CP5 electrode, which is associated with the left BA39 area. The feature is observed to be more prominent in the PSF group than in the CTF group, as it is also highly significant, but is a more frequent feature in the PSF group compared to the CTF group (in the PSF group 13 PSD_CP5 feature, in the CTF group 10 PSD_CP5). It is also notable that this feature appears first in the PSF group, whereas in the CTF group it is fourth, following the LZC_FC4, LZC_T7, and PSD_Fp1 features. However, as in the PSF group, it is associated with features deemed more and also less important by the models in the CTF group. Further investigation into the comparability of these features between the two groups to differentiate the symptoms of deficits in these areas may provide further insights into the functioning of the two disorders. The left side of BA21 (T7) is another area associated with more important features, responsible for auditory association, higher-order auditory processing, speech processing, and partly for visual association. Another electrode with more important features is at the CP6 electrode, which is associated with the right BA39 area. The BA39 region is implicated in the processes of face recognition, reading (text perception), and visuomotor orientation [65]. The areas BA10, BA9, and BA7 (Fp1, AF3, CP1) are responsible for cognitive and executive functions and also appeared in the PSF group. Lastly, BA19 (P7, P8, PO3) is responsible for object and face recognition [65]. The identification of early symptoms in bipolar disorder is a challenging endeavor, particularly given the overlap between its symptoms and those associated with schizophrenia or other psychosis spectrum disorders [37]. The findings of Payá et al. (2013) and Chan et al. (2019) indicate that individuals diagnosed with schizophrenia exhibit more pronounced cognitive deficits than those with bipolar disorder [22,38]. Both conditions are associated with substantial social adjustment challenges and impaired learning performance. From a neuropsychological perspective, aberrantly elevated amygdala function and impaired prefrontal working memory may serve as indicative markers of bipolarity [64]. However, the precise premorbid symptomatology of bipolarity remains uncharted territory, and as a result the interpretation of these findings raises several questions [37]. Further investigation is required to ascertain the significance of the involvement of these areas. Furthermore, given that bipolar disorder is a disorder in which white matter involvement is significant and considering that EEG is a tool for detecting brain surface signals, it is evident that a combination of tools and approaches may be necessary to identify precisely defined biomarkers [64]. Nevertheless, the investigation of deeper brain structures via EEG is not an unresolved issue. There have been several attempts in the literature to use other imaging modalities to gain deeper insights into how to define and correlate EEG function with other tools, even for problems such as bipolarity, and it may therefore be supposed that such data could potentially shed light on additional areas where it may be worthwhile exploring (e.g., [69,70,71,72,73,74,75,76]).

### 5.7. PSF-CTF Comparison

Figure A4 shows the features in the PSF group that are jointly considered important by the different models. Figure 14, Figure 15 and Figure 16 show the feature type, frequency, and location distribution of the important scores, and Figure 13 in shows the SHAP summary plots of the ANN(AHFS) models. A comparative analysis of the two groups revealed a salient feature localized at electrode PO4 (R. BA19), a brain region with a role in object and facial recognition [65]. Lempel-Ziv complexity metrics exhibited an elevation in the PO4 channel for the PFS group and the P4 channel for the CTF group. The functional implications of these findings, associated with the roles of right BA39 (P4) and right BA19 (PO4), suggest potential connections to the symptomatology of schizophrenia and bipolar disorder. However, based on the current state of the literature, it is hard to determine the precise possible interpretation of this result beyond this. Further data collection is necessary to gain a deeper understanding [22,59].

### 5.8. Microstates

**Figure A6:**
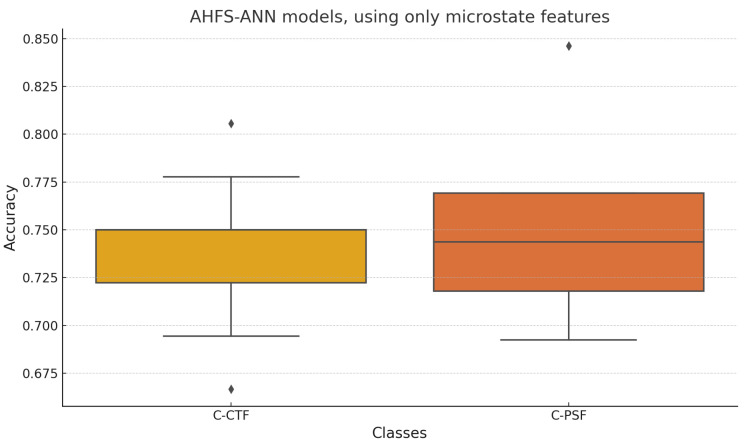
Accuracy values resulted from AHFS analysis using only microstate features. Each class has one top-performing outlier; the rest is between 70% and 75% accuracy.

Since MS features were outperformed by both feature selection methods, we conducted an additional analysis using only MS features with AHFS. The accuracy results and AHFS feature rankings can be seen in Appendix A, Figure A6 and Table A2. The accuracy of models created from these features was lower (70–75%) compared to the full feature set results (most above 85%) with AHFS, suggesting that these features may show some deviation from the control under these conditions, though not as strong as other metrics. For comparison, a previous study using microstate analysis for feature extraction successfully distinguished schizophrenia patients from healthy individuals with 84% accuracy [19]. According to the literature, bipolar disorder is characterized by a greater presence of microstate B, while schizophrenia is associated with a decreased presence of microstates A and B and a greater presence of microstate C [20,21]. Understanding the trends observed in the extracted features requires further investigation to better assess the potential applications of this methodology. Additional studies may also be needed to understand the lower accuracy, to determine whether the methodology itself contributes to the results, or if this approach has less significance in the premorbid state compared to the developed clinical condition.

### 5.9. Comparison of Results

Based on the literature, several key points should be highlighted that may provide potential directions for further investigations.

Cognitive, social, and mood changes are significant in both schizophrenia and bipolar disorder. As neurodevelopmental, chronic and in a significant proportion of cases, neurodegenerative disorders, these impairments become increasingly evident and severe over time, potentially serving as important markers, even in the premorbid stage. Most features derived from frequency analysis fell within the gamma range, which is significant in the early stages of psychotic spectrum disorders. The differentiations of gamma frequency in areas such as the prefrontal cortex and the dorsolateral prefrontal cortex are associated with higher-order cognitive functions, including inhibitory and executive functions, attention regulation, and working memory. The deterioration of these functions is reflected in gamma wave alterations and represents crucial early symptoms of psychotic spectrum disorders [36]. In both groups, significant gamma frequency features were extracted from the CP5 channel, which corresponds to BA39, specifically the angular gyrus [66]. Previous findings suggest that disruptions in the connectivity between the angular gyrus and other brain regions can lead to the cognitive impairments observed in the early stages of schizophrenia [16].

Another significant feature source was the LZC (Lempel-Ziv complexity), with a notable feature extracted from the FC5 channel in the CTF group, which is associated with BA6. Its relevance to BA6, encompassing the supplementary and pre-supplementary motor areas, is noteworthy, as this region plays a crucial role in motor control, cognitive planning, and decision-making [54,55,66]. Research indicates that individuals with psychiatric conditions such as schizophrenia often exhibit reduced LZC values, suggesting impaired neural adaptability and processing efficiency [59]. Our finding highlights a potential avenue for further investigation, offering a perspective for exploring whether or not similar deficits in motor planning and cognitive functioning are also significant in bipolar disorder.

In distinguishing between the PSF and CTF groups, the most significant features were derived from gamma frequency and Lempel-Ziv complexity in the PO4 and P4 channels, linked to BA19 and BA39, regions near the angular gyrus. This aligns with findings that differences in cognitive functioning between schizophrenia and bipolar disorder may indicate the role of the angular gyrus in the distinct cognitive profiles observed in these disorders [9]. The significance of BA39 extends to its connectivity with other brain regions involved in cognitive functions. For example, the angular gyrus is closely connected to the prefrontal cortex, which is essential for executive functions and decision-making. Disruptions in the connectivity between these regions can lead to the cognitive impairments observed in the early stages of schizophrenia [16]. This suggests that the angular gyrus, through its connections, may influence cognitive outcomes in individuals at risk for psychosis. Table 6 summarizes the literature relevance of our findings.

The potential utility of these findings is multifaceted. Firstly, the replication of the methodology and the comparison of potentially significant brain regions, electrodes, and features could provide long-term guidance on which brain areas might undergo critical changes during the premorbid phase and which symptoms should be prioritized for diagnostic and differential diagnostic purposes [16]. Secondly, in the long term, examining individual variations within similar patterns could lead to a more personalized approach to psychiatric diagnostics [6]. Further research could also offer deeper insights into schizophrenia and bipolar disorder, enhancing our understanding of how large brain networks and functionally interconnected brain regions interact. Investigating the correlations between these findings and results from other neuroimaging techniques could further refine and improve diagnostic tools, possibly making them more precise, cost-effective, and accessible [16,67,70,76].

## 6. Conclusions

Our study demonstrates that utilizing resting-state EEG-based algorithms to investigate groups at low risk for schizotypal and bipolar disorders is a promising area of research. The best models employed in our analysis achieved a remarkable 90% accuracy in distinguishing between susceptibility groups and the control group. This level of precision is comparable to that seen in patient groups at more advanced stages of these disorders.

The methods used, namely Adaptive Hybrid Feature Selection (AHFS) and Clique Forming Feature Selection (CFFS), not only enabled the selection of effective feature combinations but also facilitated a detailed examination of the intricate relationships between these characteristics. The spatial arrangement of characteristics around the electrodes in our low-risk groups displayed patterns similar to those associated with known brain impairments in advanced phases of schizophrenia and bipolar disorder.

Our approach’s effectiveness is further demonstrated by its ability to clarify the roles and impacts of specific features. This clarity enables reliable differentiation between groups, even with a limited dataset. This granular understanding of feature behavior enhances our knowledge of the distinct risk profiles and aids our insight into the underlying mechanisms of these disorders.

Given these promising results, it seems sensible to continue this line of research. Further investigation using this method should improve our understanding of the electrophysiological markers of early risk phases, potentially leading to the prevention of schizophrenia–bipolar spectrum disorders. It should enhance the accuracy of early developmental stage detection and differentiation. The microstate analysis method also has the potential to serve as an investigative tool in the study of both disorders; however, the model developed in this study did not achieve the accuracy reported in the literature. Even though our results are promising, we aim to enhance their generalizability by expanding our investigation to a larger and younger population. Identifying young individuals with premorbid cognitive dysfunctions that impact school performance is particularly important, as these difficulties reduce their chances of improving quality of life, further emphasizing the need for early detection.

### Limitations

While the small sample size raises concerns about reliability, we are confident that the identified patterns emerged from genuine brain activities. Despite employing rigorous cross-validation across all available data points, the analysis achieved high accuracy levels. Our analysis methodology was specifically designed to deliver reliable feature interpretability through noise-reduced importance scores, achieved by aggregating multiple models. Importantly, both susceptible groups showed similar deviations from the control group, as reported in the literature, based on continuous, coherent feature importance scores across multiple types of algorithm and feature groups. Despite these promising results, further validation with larger sample groups and potential longitudinal research could deliver more established, broader generalizability of these findings.

We implemented three different ML algorithms to identify various types of relations in the data. Including more alternatives could further enhance the quality of the analysis.

Data preprocessing steps included manual procedures that may introduce some level of subjectivity into the analysis. Unfortunately, we could not apply a fully automated preprocessing pipeline to deliver sufficient data quality for both microstate and frequency-based analyses. We believe the majority of interpolated or discarded segments were objectively affected by artifacts and noise. Furthermore, the ICA and resulting MS components demonstrated the desired activity patterns, and along with the stable, continuously distributed Shapley values across features (represented by a red-to-blue gradient in the summary plot), provide strong evidence that the majority of the features are derived from genuine brain signals.

Some affected regions and frequency ranges may be susceptible to muscle artifacts. Although rigorous preprocessing, especially in the MS analysis, leaves little room for such errors, it is worth mentioning this limitation.

## Figures and Tables

**Table 1:** Methods and accuracy example.

Article	Groups	Methods	Accuracy
[31]	18 BD I/20 BD II	MLP, Feature Selection (MIM, CMIM, FCBF, DISR)	82.68% (overall) 86.33% (MIM) 89.67% (CMIM) 84.61% (FCBF) 91.83% (DISR)
[32]	101 MDD/ 82 BD/81 HC	CNN	96.88%
[26]	2 datasets (age-based)	VGG-16 (CNN)	95%, 97%
[33]	14 SZ/14 C	ANFIS, SVM, ANN	100% (ANFIS) 98.89% (SVM) 95.59% (ANN)
[34]	14 SZ/14 C	CNN, LR	90% (SB) 98% (NSB)
[35]	11 SZ/20 C	kNN, LR, DT, RF, SVM	89% (SVM) 87% (RF) 86% (LR) 86% (kNN) 68% (DT)

**Table 2:** Selection criteria for participant inclusion and exclusion. In the study, the PSF group consisted of participants with O-LIFE ≥ 5, PDI-21 > 10, and TEMPS-A Cyclothymia scores < 12. This group showed higher values on the O-LIFE and PDI scales, indicating increased psychopathological risk, while the TEMPS-A scale remained within the normal range. In contrast, the CTF group had O-LIFE < 6 and TEMPS-A Cyclothymia = 11, with lower O-LIFE and PDI scores and higher TEMPS-A scores compared to the control group, suggesting emotional instability, but without a high level of psychopathological risk. The control group showed no psychopathological deviations, and their scores remained within the normal range.

Selection Criteria	Details
Initial Inclusion	University of Szeged first- and second-year students without a diagnosed psychiatric disorder.
Screening Questionnaires	TEMPS-A (Temperament), O-LIFE (Schizotypy), PDI-21 (Delusions), MDQ (Mood Disorder).
Inclusion Criteria	182 students met the screening criteria.
Exclusion Criteria	87 students excluded based on criteria, additional 2 excluded due to acute mental disorders (SCID-5).
Final Grouping	PSF Group: O-LIFE ≥ 5, PDI-21 > 10, TEMPS-A Cyclothymia < 12 (N = 30). CTF Group: O-LIFE < 6, TEMPS-A Cyclothymia total score = 11 (N = 25). Control Group: No significant psychopathology (N = 30).
Data Quality Control	Removal of participants with excessively noisy or impaired EEG data.
Final Sample Size	PSF: N = 20 (12 men, 8 women), mean age 27.66 (SD = 1.75). CTF: N = 17 (6 men, 11 women), mean age 26.82 (SD = 1.85). Control: N = 21 (9 men, 12 women), mean age 27.45 (SD = 1.89).

**Table 3:** The maximum accuracy score was obtained for the different algorithms presented in groups.

	C-CTF	C-PSF	CTF-PSF
ANN (AHFS)	0.89	0.92	0.91
ANN (CFFS)	0.79	0.71	0.62
LR	0.76	0.71	0.65
RF	0.79	0.80	0.65

**Table 4:** In the PSF group, the electrodes deemed important by the models and the brain regions associated with them are as follows: L. = Left, R. = Right, BA = Brodmann Area [66].

CP5	AF4	F8	Fp1	CP1	FC5	O2	P4
L. BA39	R. BA9	R. BA45	L. BA10	L. BA7	L. BA6	R. BA18	R. BA39
PO3	Cz	PO4	AF3	C4	CP2	FC2	O1
L. BA19	R. BA4	R. BA19	L. BA9	R. BA1	R. BA7	R. BA6	L. BA18

**Table 5:** In the CTF group, the electrodes deemed important by the models and the brain regions associated with them are as follows: L. = Left, R. = Right, BA = Brodmann Area [66].

CP5	FC5	CP6	T7	Fp1	P7	CP6	C4
L. BA39	L. BA6	R. BA39	L. BA21	L. BA10	L. BA19	R. BA39	R. BA1
FC2	F8	Cz	Fz	AF3	P8	PO3	P4
R. BA6	R. BA45	R. BA4	L. BA6	L. BA9	R. BA19	L. BA19	R. BA39
CP1	FC1	C3					
L. BA7	L. BA6	L. BA1					

**Table 6:** Key findings and their literature relevance (BA = Brodmann Area).

Key Findings	PSF Group	CTF Group	Brain Regions (Brodmann Areas)	Literature & Observations
Gamma Frequency	Features of CP5 channel	Features of CP5 channel	BA39 (Angular Gyrus)	Gamma waves linked to high-order cognitive functions [36].
Lempel-Ziv Complexity	-	Feature of FC5 channel	BA6 (Supplementary motor area)	LZC reduction in SZ suggests impaired neural adaptability [54]. Based on our findings, it may also be relevant in BD.
PSF-CTF differentiation	Features of PO4 and P4 channels	Features of PO4 and P4 channels	BA19, BA39	Angular gyrus role in cognitive differences between schizophrenia and bipolar disorder [9].

## Data Availability

The raw EEG records are available in this Huggingface repository: https://huggingface.co/datasets/nagyadam97/EEG_records_raw_schizophrenia_bipolar, accessed on 6 February 2025. The .bdf files are accessible to everyone, and the README.md file contains the class information.

## Abbreviations

The following abbreviations are used in this manuscript:

AI	Artificial Intelligence
ANN	Artificial Neural Network
AHFS	Adaptive Hybrid Feature Selection
BA	Brodmann Area
BD	Bipolar Disorder
BIS/BAS	Behavioral Inhibition and Activation System
C	Control Group
CFFS	Clique Forming Feature Selection
CNN	Convolutional Neural Network
CTF	CycloThymia Factor group
DFA	Detrended Fluctuation Analysis
DIT	Direct Information Theory
DT	Decision Trees
EASE	Examination of Anomalous Self-experiences
EEG	Electroencephalogram
ERP	Event Related Potential
fMRI	functional Magnetic Resonance Imaging
GFP	Global Field Power
HFD	Higuchi Fractal Dimension
ICA	Independent Component Analysis
LAPS	Leuven Affect and Pleasure Scale
LR	Logistic Regression
LZC	Lempel-Ziv Complexity
MDQ	Mood Disorder Questionnaire
MEQ-SA	Morningness Eveningness Questionnaire
MS	Microstates
NN	Neural Network
O-LIFE	Oxford-Liverpool Inventory of Feelings and Experiences
PDI	Peters Delusions Inventory
PSD	Power Spectral Density
PSF	Positive Schizotypy Factor Group
RF	Random Forest
rsEEG	resting-state Electroencephalogram
sampEn	Sample Entropy
SCID	Structured Clinical Interview for DSM
SVM	Support Vector Machines
SZ	Schizophrenia
TCI-R	Temperament and Character Inventory
TEMPS-A	Temperament Evaluation of Memphis, Pisa, Paris, and San Diego Autoquestionnaire

## Appendix A. Additional Figures and Tables

**Table A1:** Table of the calculated features, including their type, number, and names, as used in the machine-learning process. The microstate features are given in the first six rows, followed by the EEGlib features. The brackets contain the numerical composition of the features. The composition of the feature includes the number of feature types, the number of channels, frequency bands, and any aggregation statistics if provided.

Feature Type	Number of Features per Sample	Feature Names
Transition Matrix	16	AA, AB, AC, AD, BA, BB, BC, BD, CA, CB, CC, CD, DA, DB, DC, DD
Symmetry Test	1	symmetry_p
Markov Tests (Zero-, First-, and Second-Order)	3	markov0_p, markov1_p, markov2_p
DIT Calculations	6	prob_a, prob_b, prob_c, prob_d, dit_extropy, dit_shannon_entropy
Conditional Homogeneity Test	9	homogenity_p_{l}
Hjorth Parameters	384 (3 × 32 × 4)	hjorthActivity_{channel}_{stat}, hjorthMobility_{channel}_{stat}, hjorthComplexity_{channel}_{stat}
Power Spectral Density (PSD)	4224 (32 × 33 × 4)	PSD_{channel}_{frequency}_{stat}
Lempel-Ziv Complexity (LZC)	128 (32 × 4)	LZC_{channel}_{stat}
Detrended Fluctuation Analysis (DFA)	128 (32 × 4)	DFA_{channel}_{stat}
Engagement Level	4	engagementLevel_{stat}
Higuchi Fractal Dimension (HFD)	128 (32 × 4)	HFD_{channel}_{stat}
Sample Entropy	128 (32 × 4)	sampEn_{channel}_{stat}

**Table A2:** Summary of the AHFS analysis using only microstate features. The “Count of feature appearance” are composed of how many times the feature appeared in the top 20 models, showing that the first dozen appear in almost all of the best models. The last column is composed of which place the feature appeared; for first place the feature obtains 20 point, and for last place the feature obtains 1, so there is a maximum of 400 (20 in each run).

Microstate Features	Count of Feature Appearance	Average Point of the Feature
BC	20	365
CB	20	360
markov1_p	20	324
AD	20	296
homogenity_p_70	19	295
prob_c	20	282
prob_e	20	269
CE	20	267
DA	20	266
Shannon_hk_3	20	249
AA	20	222
prob_a	20	193
EC	18	138
symmetry_p	19	114
CD	18	111
AC	18	108
homogenity_p_65	14	98
BD	14	51
homogenity_p_40	12	51
EE	12	49
homogenity_p_45	7	25
DC	9	23
Shannon_hk_2	8	18
homogenity_p_50	5	12
Shannon_hk_4	3	7
homogenity_p_55	2	5
DB	1	1
homogenity_p_60	1	1

## Appendix B. Methodology Details

### Appendix B.1. Utilized Learning Algorithms

Three algorithms were used for the analysis:

- Logistic Regression (LR), as described by Gasso et al. (2012) [78], is a popular algorithm for binary classification tasks. In our study, we adopted LR for both feature selection and classification purposes. This algorithm was set up with an “l2” penalty metric alongside the “bilinear” solver. By fitting a linear regression model to our training dataset and then applying a logistic function, LR is able to generate probability values that aid in the classification of instances. Logistic regression offers a dependable approach for interpretable binary classification. Through the careful selection of penalty metrics and solvers, LR was efficiently utilized for feature selection and classification within our study. However, it should be mentioned that LR generally performs well with fewer features, so in the analysis it tended to select small feature sets as good ones.
- Random Forest (RF), described by Liaw et al. (2002) [79], embodies an ensemble learning technique where classification is achieved through majority voting from a collection of unpruned classification trees. These trees are developed from randomly selected subsets of the dataset, and at each decision node a randomly selected predictor determines the split, diverging from the conventional method of choosing the best split. Our implementation of RF involved the creation of 50 trees, employing the “auto” feature to cap the number of features evaluated at each split. We set the minimum number of samples required to split a node to 2, without imposing limits on the tree’s maximum depth or the maximum number of leaf nodes.
- Artificial Neural Network (ANN) sets the stage for a comparative analysis between CFFS and AHFS. Given that AHFS operates within a MATLAB framework and offers less flexibility, we endeavored to merge the neural network from AHFS into the CFFS framework. This integration met with partial success due to the inherent differences in the programming languages. The ANN is pivotal for our method comparison. The data normalization process began with the application of a min–max scaler, adjusting the dataset to a range between 0.1 and 0.9. The ANN’s structure included an input layer, succeeded by a single hidden layer with 8 neurons, in a first evaluation round; later, we found some overfitted models, so the neuron numbers were decreased to 3. During training, the bach size was 8 and early stopping was applied. This featured the sigmoid activation function and concluded with an output layer of two neurons using the softmax activation function to represent the binary classes. While the original AHFS method applied a specific optimizer, our Python adaptation used the Adam optimizer, due to the original optimizer’s incompatibility with the Python ecosystem.

Since we had small datasets, to lower the chance of over-fitting we did not tune any parameters of the models, only applying initial settings suitable for that size of data. Further details about the machine-learning algorithms can be found in the project GitHub repository “ML” folder: https://github.com/bilickiv/milabwp4/tree/akti_eeg_kodok/eeg, accessed on 6 February 2025.

### Appendix B.2. Feature Selection Details

The CFFS has the following steps:

1. Prefiltering: Due to the enormous amount of EEGlib features, The criteria to include a feature is to have at least one appear in the AHFS feature selection. Each run of the AHFS selected 20 features, and we applied 20 runs, therefore 400; in both cases, 35 unique features are selected in the process. All the microstate features were included in the feature selection.
2. Weighed graph composition with Pearson correlation: From the remaining selected features, pairwise comparisons were made to compute Pearson correlations. This resulted in a complete weighted graph, with the removal of loop and double edges.
3. Threshold-based edge deletion: Edges with weights in absolute values above a certain threshold were deleted from the graph. This threshold was 0.4 in the PSF group and 0.33 in the CTF group.
4. Clique identification: The remaining graph was analyzed to identify cliques, which are complete sub-graphs where every node is connected to every other node. A large number of cliques, approximately 5000 in both cases, were found. From these, a random selection was made of up to 600 feature sets, choosing between 3 to 9 cliques of varying sizes.
5. These cliques, identified through the feature selection process, served as the potential optimal combinations of features for training the three learning algorithms. During the learning process, Shapley values were computed, utilizing a 3-fold cross-validation approach. The resulting models were ranked according to their accuracy scores and the top 20 were selected for the next step.
6. For each model, a Shapley table was generated, consisting of columns representing the features included in the model, rows representing the individual samples, and values corresponding to the Shapley values. By aggregating the corresponding columns from each selected model, a comprehensive understanding of the model’s performance within the group was obtained. This aggregation process was conducted for models trained with each ML algorithm separately, ensuring the elimination of individual outliers and providing a robust depiction of the models’ functionality.
7. Calculation demand: Step 1 is based upon the other algorithm. Steps 2 and 3 have minuscule computing and only need to be done once for each task. Step 4, searching for cliques in the 35-node graph, takes 20–40 s (also needs to be done once). The training of the 600 model with each three algorithms takes 400–450 min (depending on the size of the feature sets)—the different algorithms take different amounts of time to train. Shapely calculation and aggregation takes 20–30 min.

For further details about the method, with examples, visit the project GitHub repository: https://github.com/bilickiv/milabwp4/tree/akti_eeg_kodok/eeg, accessed on 6 February 2025.

AHSF calculation demand:

The general runtime performance of AHFS has been illustrated in Figure 11 of Viharos et al. (2021) [23]. The computational cost varies depending on the dataset size (number of samples and features). The complete AHFS runtime, including measure precalculation, search strategy, and training, generally scales with the complexity of the dataset. Larger datasets, such as those used in wind turbine monitoring and situation detection during special machining, require significantly more computational resources, as evidenced by their placement on the figure.

For the specific runtime for the dataset utilized in this study, the AHFS framework required 197 min on average (±5 min). This runtime includes all preprocessing steps, search strategy execution, and model evaluations. These results demonstrate the feasibility of the AHFS framework for datasets of similar scale and complexity.

### Appendix B.3. Feature Type Details

The applied frequency and information theory based metrics on EEG data:

- Hjorth Parameters (Activity, Mobility, and Complexity) are computed in order to offer valuable insights into the variance, frequency attributes, and complexity of an EEG signal. Activity at Hjorth quantifies the variance, which indicates the overall strength of the signal. Hjorth mobility provides information regarding the frequency dynamics of a signal by measuring its mean frequency. Changes in frequency are utilized to quantify Hjorth complexity, which indicates the irregularity and complexity of the signal. After calculating these parameters for each of the 32 EEG channels, three features are produced per channel per chunk.
- Power Spectral Density (PSD) estimates the frequency-dependent power distribution of the EEG signal. PSD is computed within the interval of 0 to 64 Hz using a 2 Hz step size in our analysis, yielding 33 frequency bins.
- Sample Entropy (sampEn) of the EEG signal quantifies its irregularity and complexity. It measures the probability that analogous signal patterns will persist at a subsequent juncture. This attribute is computed for every single one of the 32 EEG channels.
- Lempel-Ziv Complexity (LZC) is a metric that quantifies the complexity of an EEG signal by assessing the quantity of unique patterns present in the signal. It is computed for every one of the 32 EEG channels.
- Detrended Fluctuation Analysis (DFA) is utilized to detect long-range correlations in the EEG signal. It is determined for every single one of the 32 EEG channels.
- Level of Engagement is an index that measures the degree of attention or engagement in accordance with the EEG signal. In contrast to the remaining features, engagement level generates a singular engagement score per interval by aggregating multiple features from all channels.
- Higuchi Fractal Dimension (HFD) is a method utilized to approximate the fractal dimension of an EEG signal, which serves as an indicator of the signal’s complexity. This attribute is computed for every single one of the 32 EEG channels.

The project’s GitHub repository can provide further details about the exact feature calculating codes: https://github.com/bilickiv/milabwp4/tree/akti_eeg_kodok/eeg, accessed on 6 February 2025.
